# Hereditary Diffuse Gastric Cancer: Multidisciplinary Case Report with Review of the Literature

**DOI:** 10.4061/2011/845821

**Published:** 2011-02-06

**Authors:** Rebecca Wilcox, Melody Perpich, Amy Noffsinger, Mitchell C. Posner, Kumarasen Cooper

**Affiliations:** ^1^Surgical Pathology Department, University of Vermont/Fletcher Allen Hospital, EP2-107 111 Colchester Avenue Burlington, VT 05401, USA; ^2^Department of Cancer Genetic Counseling, Creticos Cancer Center, Advocate Health Care, 901 West Wellington Avenue, Chicago, IL 60657-6708, USA; ^3^Gastrointestinal Surgical Pathology Department, Caris Life Sciences, 9978 Washington Street, Camp Dennison, OH 45111, USA; ^4^General Surgery Section, The University of Chicago Medical Center, 5841 S. Maryland Avenue, MC 5031 Chicago, IL 60637, USA

## Abstract

Hereditary diffuse gastric cancer (HDGC) is a rare, inherited cancer syndrome with at least one fourth of HDGC patients having an autosomal dominantly inherited mutation of *CDH1* (*E-Cadherin*). Penetrance is relatively high (70–80% lifetime risk for gastric cancer). It is important for pathologists to recognize the syndrome's phenotype in early gastric lesions: patchy intramucosal signet ring cells often associated with pagetoid spread. Due to the insidious nature of this lesion, surveillance is limited and currently prophylactic gastrectomy is an option chosen by many HDGC patients. We present a case report from a multidisciplinary team of authors with a review of the literature that includes the updated guidelines for *CDH1* genetic testing.

## 1. The Case 

**Figure 1:**
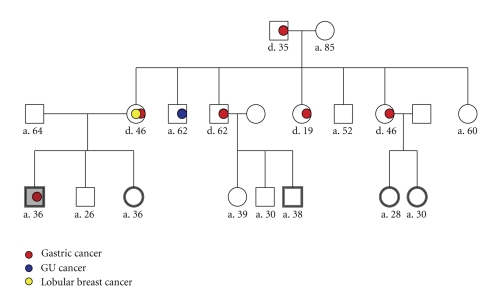
Pedigree of case study (index) patient (shaded grey). Note that five additional family members show a history of gastric cancer with the youngest presentation being 19 years of age. Double outline indicates positive genetic testing for E-Cadherin mutation (1212delC).

An asymptomatic 36-year-old male presented for an elective, increased risk, upper endoscopy (EGD). The patient's remarkable history included at least five family members diagnosed with gastric carcinoma, four known to have occurred at an early age (Figure 1). He had a documented mutation in the *CDH1* (*E-Cadherin*) gene (1212delC) identified approximately one month prior to this surveillance EGD. The EGD was essentially normal. The stomach insufflated and decompressed without difficulty and there was no visual evidence of mucosal changes. EGD biopsies were read as gastric body mucosa without diagnostic abnormality and were negative for *Helicobacter pylori*, intestinal metaplasia, dysplasia, and malignancy.

Three months later, following genetic counseling, the patient was admitted for an exploratory laparotomy with a prophylactic total gastrectomy with D2 lymphadenectomy and end-to-side Roux-en-Y esophagojejunostomy and needle catheter feeding jejunostomy. The patient's sister and two cousins had recently undergone similar prophylactic procedures.

**Figure 2:**
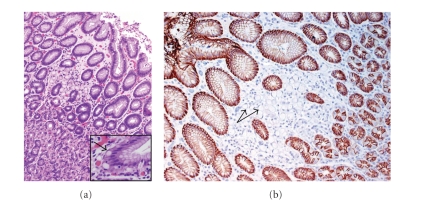
(a) Focal intramucosal signet-ring cell adenocarcinoma in case study patient with known CDH1 mutation. Insert highlights area of pagetoid spread. (b) Focal absence of E-Cadherin staining highlights the tumor cluster in case study patient. In those HDGC cases in which tumor cells show decreased E-cadherin expression, the normal background epithelium serves as an excellent internal control.

Surgical exploration showed no evidence of metastatic disease. Gross pathological examination of the stomach revealed intact, pink mucosa with normal rugae and no masses. The entire surgical margins, multiple (>40) gastric sections from all anatomic zones, and the entire perigastric adipose tissue were submitted for histologic review. Histological examination revealed multifocal, intramucosal signet-ring cell adenocarcinoma with focal pagetoid spread of the signet ring cells in preserved fundic glands (Figure 2(a)). The sections were negative for gastric dysplasia and *Helicobacter pylori*. All margins and lymph nodes were negative for tumor (AJCC pathologic stage pT1a pN0).

The patient had an uneventful postoperative recovery with an upper gastrointestinal study done on postoperative day #5 that showed no anastomotic leaks or strictures. He was discharged on postoperative day #7. Followup examinations revealed extensive weight loss (approximately 50 lbs) but the patient was otherwise doing well.

## 2. Molecular Carcinogenesis

Only a small number, 2–8%, of all gastric carcinomas arise from inherited gastric cancer syndromes [1, 2]. The majority of families with autosomal dominant familial gastric carcinoma will have the diffuse, poorly differentiated (linitis plastica) morphologic subtype and are referred to as *hereditary diffuse gastric cancer *(HDGC). A germline mutation in the tumor suppressor gene *CDH1* (*E-Cadherin*) is identified in approximately 25–48% of individuals with HDGC [3, 4]. E-Cadherin is an epithelial cell-cell adhesion molecule essential to cell differentiation and normal epithelial cell architecture. It is therefore not surprising that germline mutations in *E-Cadherin* are highly specific for families who meet HDGC criteria and has not been described in families with inherited intestinal type/morphology gastric carcinoma [3, 5, 6]. 

In 1999, the International Gastric Cancer Linkage Consortium (IGCLC) met to first define criteria required to select those patients appropriate to receive mutational screening for HDGC [5]. Strict application of these 1999 IGCLC criteria found approximately one third of screened patients to be heterozygous for a germline, point or frameshift, mutation of the *CDH1* gene on chromosome 16q22.1 [6, 7]. This implies that there are either currently unidentifiable *CDH1* mutations or other genes causing HDGC in some families. Medical management recommendations are particularly difficult in these families with undetectable *CDH1 *mutations. There are at least twenty-seven documented inactivating (truncating) *CDH1* mutations scattered at various exons along the *E-Cadherin* gene described in a diverse ethnic population [3, 8–10]. In a recent study addressing patients that met HDGC criteria but lacked *CDH1* germline point mutations, Oliveira et al. found that 6.5% of their study patients had large deletions affecting the *CDH1* locus by using multiplex ligation-dependent probe amplification (MLPA) [7]. Therefore, analysis for large genomic deletions using alternative techniques such as MLPA or array comparative genomic hybridization (CGH) should be explored in highly suspicious cases in which standard DNA sequencing is negative for point mutations.

The current hypothesis for how HDGC susceptibility cancers lose their CDH1 heterozygosity and thus their E-Cadherin expression follows the “two-hit” mutation theory [11]. Hypermethylation of the *CDH1* promoter is the most common cause of inactivation of the second allele; however, mutation and loss of heterozygosity (LOH) are also well-documented culprits [11–13]. Interestingly, the mechanisms of the “2nd hit” may differ in primary tumors versus metastases, an important consideration for therapeutics. The morphologic expression of the second hit is multifocal clusters of tumor cells that have lost or show abnormal (reduced) E-cadherin expression (Figure 2(b)). The subsequent step, progression to invasion of the submucosa, is less well defined. The current line of thought involves an integrated role between additional genetic events and changes in microenvironment. Unfortunately, there are no current means of predicting the time course between tumor expansion and submucosal invasion. 

The *CDH1 *mutation carries a high penetrance with carriers of germline mutations having an estimated lifetime risk of 67% in men and 83% in women to develop gastric carcinoma [4, 14]. In HDGC families, the most commonly described malignancy other than diffuse gastric carcinoma is lobular breast adenocarcinoma. Female carriers of a germline *CDH1* mutation have a 40% lifetime risk of developing this subset of breast cancer [14]. In general, susceptibility cancers in HDGC occur at a relatively early age, with the average age of presentation of diffuse gastric carcinoma being 38 years [15].

## 3. Clinicopathologic Management 

The IGCLC guidelines were first developed in 1999 to support clinical management of families felt to be predisposed to gastric carcinoma [5]. This multidisciplinary group has recently provided us with an updated, somewhat broadened version of their original guidelines as to which patients should be offered molecular genetic testing (Table 1) [4]. Any patient who meets the minimum requirements for HDGC listed in Table 1 should be offered genetic counseling and testing for the *CDH1* mutation. Health care professionals experienced in cancer genetics must provide those patients that choose to undergo this testing pre- and posttesting genetic counseling. 

In general, annual endoscopy using a white light high-definition endoscope is recommended for surveillance of HDGC patients. Using histopathology mapping in six gastrectomy specimens from New Zealand Maori HDGC families, Charlton et al. showed a preferential pattern of intramucosal diffuse gastric carcinoma in the body-antral transitional zone [16]. Based on their study, they proposed a targeted approach to endoscopy with the goal of minimizing sampling error. However, results from prophylactic gastrectomy specimens from other areas of the world did not show similar findings [17–21]. Due to the discrepancy in study results regarding localization of early gastric lesions, multiple gastric biopsies representing all of the major gastric anatomic zones is recommended. The HDGC endoscopic protocol provided by the most recent consensus guidelines suggests the following biopsies: a single antral biopsy taken first for surveillance of *H. pylori* status, any focal lesions, and in addition at least three biopsies from each anatomical area (prepyloric area, gastric antrum, transitional zone, gastric body, gastric fundus, and gastric cardia) [4]. This extensive sampling is driven by the insidious phenotype of this disease (patchy, diffuse growth pattern of gastric carcinoma under endoscopically normal gastric mucosa), which leads to low sensitivity in currently available surveillance procedures.

Our HDGC patient was found to have occult intra-mucosal signet ring cell adenocarcinoma on resection despite having a normal endoscopic appearance to the stomach and no diagnostic findings by random endoscopic biopsies. Upon review of the literature representing different, independent HDGC families, the identification of indolent gastric cancer is consistent with the pathology found in the majority of cases following prophylactic gastrectomy [19, 20, 22]. In light of the inherent limitations to the current screening procedures/tests available for HDGC patients, a prophylactic gastrectomy is not only a reasonable option but some may argue a life saving one. The estimated 30-day postgastrectomy mortality rate is cited as 3–6% [23] and most likely reaches even smaller numbers when a gastrointestinal surgeon who routinely performs gastrectomies and other major surgeries performs the procedure. To put this in perspective, the 5-year mortality rate in patients with symptomatic, invasive gastric carcinoma is 90% [24]. 

However, this procedure is not without long-term complications. All patients who undergo gastrectomy will have postoperative weight loss. Many will have metabolic complications including malabsorption, diarrhea, and/or “dumping syndrome.” Dieticians are often required to assist postsurgical patients in nutritional management. There are also secondary surgical complications such as esophageal strictures. A more thorough understanding of the long-term physical and psychological effects of this surgery will only emerge through followup of these relatively young HDGC patients. The current consensus guidelines have called for a central registry of HDGC patients who have undergone prophylactic gastrectomies [4]. This would provide essential prospective data regarding effects of the surgery as well as long-term followup regarding disease-free status.

Prophylactic gastrectomies for HDGC will most likely occur at major academic institutions in which specialized gastrointestinal pathologist are readily available. The gastric specimen is inked and fixed with lymph node retrieval as in any gastrectomy specimen. However, following adequate (overnight) fixation, these specimens should be photographed with mapped sampling occurring from all anatomical zones as well as submission of the entire surgical margins. If carcinoma is not identified, additional mapped sampling will be required. The focal intramucosal signet ring cells are generally identified on H & E morphology. A combination of cytokeratin (positive immunoreactivity) and E-Cadherin (negative or reduced immunoreactivity; see Figure 2(b)) can highlight these foci, especially in areas of pagetoid spread. Interestingly, in their study of eight total gastrectomy specimens done for germline *E-cadherin *mutations, Rogers et al. found two cases which showed reversion of E-cadherin expression in foci of deeply invasive adenocarcinoma while the superficial signet ring cells cancer showed the expected loss or reduced E-cadherin expression [21]. Additional, nonspecific histologic features described in some cases of HDGC include foveolar hyperplasia with or without tufting, cytoplasmic vacuolization, and clustered histiocytes or vaguely granulomatous reactions occurring around ruptured glands [4, 18, 21]. 

The IGCLC also suggests that female carriers of the *CDH1* mutation receive high-risk screening for lobular breast adenocarcinoma from the age of 35. Other cancers, such, signet ring cell carcinoma of the colon, have been proposed to be associated with HDGC. Colon cancer screening may therefore be recommended in HDGC patients who have a pedigree showing colon carcinoma presenting before the age of 40. This type of personalized heightened cancer screening emphasizes the need for thorough documentation of family histories as well as the importance of enrolling HDGC patient into cooperative registries for ongoing clinical research.

## 4. Conclusion

At least one third of all patients with HDGC have an autosomal dominantly inherited mutation of the *CDH1* (*E-Cadherin*) gene. Penetrance is high with a specific phenotype of diffuse, signet ring cell morphology. Pathologists should be aware of this phenotype of patchy clusters of intramucosal signet ring tumor cells associated with a pagetoid spread of individual signet ring cells (Figure 2), although not known to be entirely specific, this finding, especially in a young patient, may warrant a discussion with the treating clinician regarding referral to a genetic counselor. In patients known to have HDGC, a multidisciplinary approach, including genetic counselors, subspecialized pathologists, clinical researchers, dietitians, and experienced gastrointestinal surgeons, is essential in the management of these patients. Due to the insidious nature of early lesions in this disorder, endoscopy is not an adequate screening method. However, annual white light high-definition endoscopy with extensive biopsy sampling may provide a method of surveillance for those patients who are not good surgical candidates, who refuse more aggressive methods, or are carriers of mutations (e.g., missense) where clinical significance is less defined.

The prophylactic gastrectomy specimen of our patient known to carry a *CDH1* mutation was found to have multifocal intramucosal signet ring cell adenocarcinoma despite normal endoscopic exam and biopsies. Previous publications, of at least three major groups, document the same finding in multiple HDGC families, making these risk-reducing gastrectomies simultaneously therapeutic. Currently there is no way of determining the latent period between the intramucosal CDH1 −/− adenocarcinoma and invasion into submucosa. However, it is important to note that our patient had at least two family members that died of HDGC at ages younger (19 and 35 years of age) than his age at the time of gastrectomy (36 years of age). Until better screening tests emerge, gastrectomy may be the only adequate means of lengthening survival in carriers of the *CDH1* mutation.

## Figures and Tables

**Table 1:** (Updated) Criteria for CDH1 molecular genetic testing.

Two or more cases of gastric cancer in a family, with at least one histologically confirmed diffuse gastric cancer (DGC) diagnosed before the age of 50.
Three or more confirmed cases of DGC in 1st or 2nd degree relatives, independent of age of onset.
An individual diagnosed with DGC before the age of 40.
An individual or family members diagnosed with DGC and lobular breast cancer, one being diagnosed before the age of 50.
